# Rational Design and Efficacy Evaluation of a Novel Solid Dispersion-Based Bempedoic Acid–Ezetimibe Fixed-Dose Combination Tablet Versus Nexlizet^®^

**DOI:** 10.3390/pharmaceutics18050580

**Published:** 2026-05-07

**Authors:** Mohamed Heikal, Wael Ali, Mahmoud A. Mahdy, Eman Gomaa

**Affiliations:** 1 Department of Pharmaceutics, Faculty of Pharmacy, Zagazig University, Zagazig 44519, Egypt; waelali40@gmail.com (W.A.); mahmoudabdelghanymahdy@yahoo.com (M.A.M.); eman_pharmaceutics@yahoo.com (E.G.); 2 Safety Science Medical Company (SSMC), FAS Group, Riyadh 12382, Saudi Arabia; 3 Nanotechnology Research Center (NTRC), The British University in Egypt (BUE), Cairo 11837, Egypt

**Keywords:** bempedoic acid, ezetimibe, Nexlizet^®^, solid dispersion, film-coated tablet, in vitro evaluation, lipid profile

## Abstract

**Background/Objectives**: For hyperlipidemic patients with statin resistance, a fixed-dose combination of a non-statin drug such as ezetimibe (EZT) and bempedoic acid (BA) provides a significant benefit. Although both drugs exhibit poor aqueous solubility, the oral bioavailability of EZT is more critically limited by dissolution, whereas BA maintains adequate absorption due to its high intestinal permeability. With regard to the reference product, Nexlizet^®^ (180/10 mg), our study focused on developing a novel tablet with superior in vitro performance without incorporating sodium lauryl sulfate (SLS), as it may potentially alter BA absorption. **Methods**: The solid dispersion technique (co-precipitation) was applied using the Kollidon^®^ VA64 polymer, and the solid state was characterized through differential scanning colorimetry (DSC), X-ray powder diffraction (XRPD), and Fourier transform infrared spectroscopy (FTIR). The prepared solid dispersions (SDs) were formulated into film-coated tablets (FCTs) and were characterized physically and for drug performance, and an animal model study was also conducted. **Results**: The solid-state analysis of the optimized SD formula (S30) revealed reduced drug crystallinity with no drug–carrier chemical interaction. The optimized formula (F30), a film-coated tablet, successfully achieved comparative in vitro dissolution versus Nexlizet^®^ and passed the accelerated stability study. Furthermore, in vivo evaluation revealed that F30 significantly reduced serum total cholesterol (TC), triglycerides (TG), and low-density lipoprotein (LDL), with an increase in high-density lipoprotein (HDL), in an animal model. **Conclusions**: These findings confirm that the SD technique is an effective one-step approach to co-formulating both APIs, simplifying manufacturing processes and optimizing the batch size.

## 1. Introduction

Dyslipidemia is a prevalent metabolic disorder marked by abnormal blood lipid profiles, primarily involving cholesterol, low-density lipoprotein (LDL), high-density lipoprotein (HDL), and triglycerides (TG). Over time, having unhealthy levels of blood fats can lead to fatty deposits building up inside the arteries, a condition called atherosclerosis, which increases the risk of heart problems. Management typically involves lifestyle changes, i.e., regular exercise and dietary adjustment, along with lipid-lowering medications—most commonly statins [1].

Statin drugs are used to treat and manage hypercholesteremia via the selective and competitive inhibition of the hydroxy methyl glutaryl-CoA (HMG-CoA) reductase enzyme, which reduces TG, LDL, and total cholesterol levels while increasing HDL levels [2,3]. Statins are generally regarded as well-tolerated medications. Statin intolerance is defined as any adverse event or abnormality not accepted by the patient and leading to the discontinuation of therapy [4,5]. Typically, musculoskeletal symptoms represent the predominant cause of therapy discontinuation, whereas laboratory abnormalities are comparatively infrequent [5,6,7]. Apart from statin-associated muscular symptoms (SAMS), additional side effects of statin therapy that may impair patient quality of life include erectile dysfunction, headache, nausea, dyspepsia, and alopecia [5,8]. Patients who are intolerant to statins are advised to switch to non-statin drugs.

**Figure 1:**
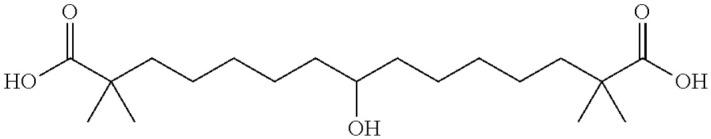
Chemical structure of bempedoic acid.

Bempedoic acid (BA) is known chemically as 8-hydroxy-2,2,14,14-tetramethyl-pentadecanedioic acid. Its CAS number is [738606-46-7] and its molecular weight is 344.5 g per mole, and its chemical formula is C_19_H_36_O_5_. It is a white to off-white crystalline powder that is insoluble in water and aqueous solutions below pH 5 and highly soluble in ethanol, isopropanol, and pH 8.0 phosphate buffer. The structural formula is shown in Figure 1 [9].

**Figure 2:**
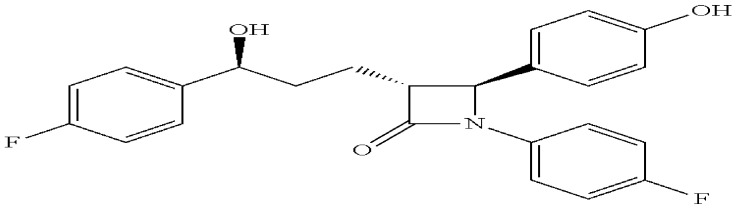
Chemical structure of ezetimibe.

Ezetimibe (EZT) is known chemically as 1-(4-fluorophenyl)-3(R)-[3-(4-fluorophenyl)-3(S)hydroxypropyl]-4(S)-(4-hydroxyphenyl)-2-azetidinone. Its CAS number is [163222-33-1] and its molecular weight is 409.4 g per mole, and its molecular formula is C_24_H_21_F_2_NO_3_. It is a white, crystalline powder that is practically insoluble in water and freely to very soluble in ethanol, methanol, and acetone. The structural formula is shown in Figure 2 [9].

Nexlizet^®^ tablets were approved in the US in 2020 and are manufactured by the company Esperion Therapeutics, Inc. In addition to 180 mg of BA and 10 mg of EZT as active ingredients, each film-coated tablet also comprises many other inactive ingredients [10,11]. Bempedoic acid is a first-in-class adenosine triphosphate citrate lyase (ACL) inhibitor. It acts to reduce cholesterol production by blocking the ACL enzyme in the cholesterol biosynthesis pathway [11]. Ezetimibe acts by inhibiting the Niemann–Pick C1-like 1 (NPC1L1) transporter protein in the brush border of the small intestine; as a result, it prevents the absorption of dietary and biliary cholesterol into the bloodstream [12]. It is recommended as a dietary supplement and can be beneficial for adults who exhibit maximum tolerance to statin therapy. In Nexlizet^®^, sodium lauryl sulfate (SLS) is included as a surfactant to resolve the solubility issues of EZT, since EZT has poor water solubility beyond the physiological pH. Despite being pH-dependent, BA does not require a surfactant because its greater solubility is reached at a high (intestinal) pH, ensuring in vivo absorption and dissolution. Due to the distinct physicochemical properties of BA (poor flowability and stickiness), and considering that the potential impact of SLS on the dissolution behavior of BA remains unclear, a formulation strategy was adopted to avoid possible interference; the formulation was prepared as both monolayer and bilayer tablets. For the monolayer tablet, each active ingredient was granulated separately, mixed, compressed into a single layer, and coated. For the bilayer tablet, each active ingredient was prepared as two distinct layers, compressed into a bilayer tablet, and coated [9]. Nexlizet^®^ contains the following inactive ingredients: lactose monohydrate, magnesium stearate, microcrystalline cellulose, sodium lauryl sulfate, sodium starch glycolate, hydroxypropyl cellulose, colloidal silicon dioxide, and povidone K30. The film coating material, Opadry^®^ AMB II, includes titanium dioxide, sodium lauryl sulfate, partially hydrolyzed polyvinyl alcohol (PVA), glyceryl monocaprylocaprate, FD&C Blue #1/Brilliant Blue FCF Aluminum Lake, FD&C Blue #2/Indigo Carmine Aluminum Lake, and talc [9].

According to the BCS, APIs are classified based on their solubility and permeability into four classes [13,14]. BA and EZT are considered BCS Class II drugs of low solubility and high permeability. Their low solubility and slow dissolution in gastrointestinal fluids (acidic conditions) constitute the primary barriers to oral bioavailability. Therefore, improving their solubility and dissolution rates is crucial for their in vivo efficacy [13,15,16].

Solid dispersion is a strategy for drug solubility enhancement. In this approach, the drug can be distributed molecularly through a carrier matrix as amorphous particles (clusters) or in crystalline particles [17,18].

Relative to conventional methods such as spray drying or hot-melt extrusion, the co-precipitation method offers several distinct advantages, including simplicity, cost-effectiveness, and scalability, making it highly suitable for both laboratory-scale production and industrial manufacturing.

Nexlizet^®^ presents significant formulation challenges relative to its physicochemical and biopharmaceutical characteristics. While BA is characterized by poor flowability and a sticky nature, EZT exhibits low aqueous solubility and dissolution-limited absorption, necessitating complex manufacturing approaches such as separate granulation. These constraints not only increase the process complexity but also limit the formulation flexibility and optimization potential; for example, the blending of separately processed granules may increase the risk of segregation arising from disparities in particle size distribution, density, and flow characteristics, potentially compromising content uniformity. In addition, variations in granule layer size may further complicate batch size determination and scale-up, introducing potential challenges in maintaining process consistency and reproducibility during the manufacturing of fixed-dose combinations of BA and EZT.

This study is the first to report a surfactant-free fixed-dose combination of BA/EZT (180/10 mg) prepared using a co-precipitation-based solid dispersion approach that was intentionally employed to induce the amorphization of both BA and EZT within a single composite system prior to tablet formulation—unlike Nexlizet^®^ (180/10 mg), which uses an anionic surfactant (SLS) to resolve the solubility issue of EZT.

Although BA exhibits high oral bioavailability, the application of solid dispersion in this study was primarily intended to improve its physicochemical performance and constrain the potential risk within the fixed-dose combination excipients (SLS), rather than to enhance its absorption.

The proposed formula achieves rapid dissolution with a release profile comparable to Nexlizet^®^ but without the use of SLS (SLS-free), which potentially improves formulation robustness, regulatory acceptability, and patient tolerability. In addition, this study overcomes the gelling effect associated with tablet-based solid dispersion. Accordingly, this study demonstrates an alternative manufacturing process to wet granulation and provides a unique formula with mechanical integrity, robustness, and downstream process suitability, including friability and coating performance. It passes the accelerated stability test and proves its antihyperlipidemic efficacy in a hyperlipidemic rat model with an improved lipid profile.

## 2. Materials and Methods

### 2.1. Materials

Bempedoic acid [Lee Pharma Limited, Telangana, India, purity of 99.6%]; ezetimibe [Glenmark Life Sciences Ltd., Maharashtra, India, purity of 99.3%]; copovidone, also known as PVP VA64 (Kollidon^®^ VA64); crospovidone (Kollidon^®^ CL); crospovidone (Polyplasdone^®^ XL10); microcrystalline cellulose (Avicel^®^ PH 102); colloidal silicon dioxide (Aerosil^®^ 200); magnesium stearate; and Opadry^®^ AMB II were obtained as gift samples from Future Pharmaceutical Industries (FPi), Badr City, Egypt. Nexlizet^®^ 180/10 mg FCTs (lot number: 1750787) were purchased from the market. All other chemicals were of high analytical grade.

### 2.2. Methods

#### 2.2.1. Preparation of the Main Solid Dispersion

The main solid dispersion (SD) of BA/EZT was prepared by co-precipitation (co-evaporation) using Kollidon^®^ VA64 as a carrier. The preparation of the main SD part (S1 to S16) according to different polymer-to-API ratios is illustrated in Table 1. Briefly, the carrier was accurately weighed and then dissolved in a sufficient volume of purified water in a porcelain dish (aqueous solution). The two APIs were dissolved in a minimum volume of absolute ethanol as a common organic solvent. The aqueous carrier solution and the organic solution of drugs were mixed via continuous stirring for 10 min using a BOECO Magnetic Stirrer (MSH 140, Hamburg, Germany) [18,19].

For solvent evaporation, the suspension was subjected to a Memmert vacuum oven (VO29, Schwabach, Germany) and left to dry for 12 h at 45 °C [9]. The dried precipitates were milled using a mortar and pestle, sieved through a #40 mesh [20], and then kept in a desiccator at ambient temperature.

This formulation (S1 to S16) represents a systematic formulation design approach aimed at systematically evaluating the influence of key formulation and process variables (polymer ratio) on the performance of the solid dispersion system. The formulations were grouped into four series based on the polymer-to-API ratio (S1 to S4, S5 to S8, S9 to S12, and S13 to S16), which led to a change in the SD weights of the different formulations. The outcomes of these series guided the rational optimization and subsequent formulation modifications.

#### 2.2.2. Preparation of the Main Film-Coated Tablets Containing the Main Solid Dispersion

All prepared main SD formulations (S1 to S16) were incorporated into film-coated tablets (FCTs) (F1 to F16) by the direct compression method. Briefly, Kollidon^®^ CL served as a disintegrant (5–20%), while Avicel^®^ PH 102, Aerosil^®^ 200, and magnesium stearate served as a filler, glidant, and lubricant at fixed concentrations of 15%, 1%, and 1%, respectively, as shown in Table 2.

The change in tablet weight among the different formulations was due to intentional changes in polymer and disintegrant ratios during formulation optimization. The polymer and disintegrant ratios were the primary variables, while the relative proportions of the remaining excipients were kept constant across formulations. Therefore, the observed differences in tablet properties are mainly attributed to these controlled variables.

First, Kollidon^®^ CL and Avicel^®^ PH 102 were sieved at mesh size 450 µm (#40 mesh), and the prepared SD was added. Aerosil^®^ 200 was also sieved at mesh size 250 µm (#60 mesh) and added to the previous blend. After the blend was mixed for 10 min [21], magnesium stearate was sifted at mesh size 250 µm (#60 mesh) and added and mixed with the blend for 5 min [21].

The final blend of each formula was directly compressed into a tablet using a single tableting machine (ERWEKA, GmbH Type EP-1, Heusenstamm, Germany). Formulae F1 to F14 were compressed with a plain punch measuring 11.0 mm on both sides, while formulae F15 to F16 were compressed with a plain, oblong, biconcave punch measuring 18.0 mm on both sides.

Core tablets were coated with Opadry^®^ AMB II (12–15% aqueous solution) to achieve 2–3% weight gain using a SHAKTI coating machine (SHAKTI, Ahmedabad, India) [22]. All formulations were coated prior to evaluation because the coating layer can significantly affect the disintegration time and dissolution behavior. Applying coatings to all formulations ensured a fair comparison with the commercial coated Nexlizet^®^ tablets and prevented bias in assessing the effects of the solid dispersion and formulation variables.

#### 2.2.3. Evaluation of the Main Film-Coated Tablets

##### Pre-Compression Evaluation

1. Powder flowability

Powder flowability is a pivotal parameter in the tablet manufacturing process. Different methods were used in assessing the flow properties, including the angle of repose, compressibility index (*CI*), and Hausner’s ratio.

2. Angle of Repose

The angle of repose (θ) was evaluated using the fixed funnel method, through which the powder blend was allowed to fall freely. The funnel (6.8 cm diameter) was fixed with its lower tip (8 mm diameter) at 2.5 cm over a piece of graph paper. The heap radius and the corresponding angle of repose were determined. The angle of repose (θ) was estimated using the following formula:

$$Tan\theta =\frac{h}{r}$$

in which θ is the angle of repose, “h” is the height of the cone, and “r” is the radius of the cone base.

3. Hausner ratio and compressibility index

Bulk density was assessed by determining the volume occupied by a pre-weighed powder sample in a 50 mL graduated cylinder. The tapped density was measured using a standardized tap density apparatus operated for (100, 500, 1750, 1250) taps over 5 min at a stroke height of 20 mm (100 strokes per minute). The difference in tapped volume beyond 500 taps was minimal (<2%), indicating effective stabilization. Therefore, 500 taps were considered sufficient for assessing powder flowability and compressibility [23].

The Hausner ratio and compressibility index (*CI*) were then calculated using the following equations [21,24], respectively:

$$Hausner\;ratio=\frac{Tapped\;density}{Bulk\;density}$$

$$Compressibility\;index\;\left(CI\right)=\frac{\left(\;Tapped\;density-Bulk\;density\;\right)}{Bulk\;density}\times 100$$

##### Post-Compression Evaluation

1. Physical evaluation

- Disintegration time

The disintegration time (D.T.) was measured according to the Ph. Eur. 10.0 procedures using a disintegration tester (ERWEKA ZT 120, Heusenstamm, Germany) with 800 mL of purified water maintained at 37 ± 0.5 °C. One tablet was placed in each basket, and the D.T. was recorded in seconds (s) when no residue of the original tablet remained above the basket mesh [24].

- Weight variation

Weight variation was assessed by weighing 10 tablets individually using an electronic balance (METTLER TOLEDO, MS204S, Greifensee, Switzerland) [21] due to the small batch size (~100 tablets) during the formulation development stage.

- Hardness

Tablet hardness was measured using an ERWEKA hardness tester (TBH 125D, Heusenstamm, Germany) by measuring the diametrical force required to fracture a single tablet placed between the moving plates [25], and the used unit was kiloponds (KP).

- Diameter and thickness

The diameter and thickness of the prepared tablets were measured using a digital caliper (Vogel, Kevelaer, Germany) [24].

- Friability

Friability was determined on tablets equivalent to 6.5 g in an ERWEKA friability tester (ERWEKA, tar II, Heusenstamm, Germany) [21]. As the friabilator rotates, tablets are tumbled and dropped 100 times from a fixed height. After dedusting, the weight loss is measured, with a reduction of less than 1% considered acceptable for uncoated compressed tablets [26].

2. Drug performance

- Drug content

The drug content of the different formulae was determined by dissolving one film-coated tablet in a 50 mL volumetric flask, to which exactly 25 mL of a diluent (acetonitrile–water, 50:50% *v*/*v*) was added, and the mixture was sonicated for 10 min at room temperature using a Bandelin Sonorex RK102H ultrasonic bath (Bandelin Sonorex, Berlin, Germany). The volume was completed with the diluent to reach 50 mL. A 5 mL aliquot was further diluted with the mobile phase (acetonitrile–phosphate buffer pH 5, 45:55 *v*/*v*). Quantification was performed using an Agilent HPLC 1260 Infinity II (Agilent Technologies, Santa Clara, CA, USA) with a photodiode array detector at 210 nm, and results were expressed as the mean ± SD of six replicate measurements [27].

Drug content was calculated based on the ratio of the HPLC peak area of the sample and a standard solution, in accordance with standard chromatographic practices described in the USP <621> and ICH Q2(R2) guidelines, according to the following equation:

$$Drug\;content\;\left(\%\right)=\frac{Area\;(test)}{Area\;(standard)}\times \frac{Concentration\;\;(standard)}{Concentration\;(test)}\times \frac{Purity}{100}\times \frac{\left(100-water\;content\right)}{100}\times 100$$

- Drug dissolution

The drug dissolution profiles of all formulations were evaluated against Nexlizet^®^ using USP apparatus II(paddle stirrer, Erweka, Heusenstamm, Germany), in accordance with the FDA-recommended dissolution media and sampling intervals for BA/EZT FCT. Each tablet (180 mg of BA and 10 mg of EZT, *n* = 6) was tested in dissolution medium: BA (900 mL, 0.05 M phosphate buffer, pH 6.6, under a stirring rate of 50 rpm at 37 ± 0.5 °C) and EZT (900 mL, 0.05 M sodium acetate buffer, pH 4.5 with 0.45% SLS, under a stirring rate of 50 rpm at 37 ± 0.5 °C). A sample of 10 mL was withdrawn at pre-determined time intervals (10, 15, 20, 30, and 45 min) [28], and an equivalent volume of dissolution medium was added to maintain the sink condition. The two drugs were quantitatively assessed using an Agilent HPLC 1260 Infinity II (Agilent Technologies, Santa Clara, CA, USA) with a photodiode array detector at 210 nm [27]. Results were expressed as the mean ± SD using six replicates.

The percentage of drug dissolution at each time was calculated according to the following equation:

$$\begin{array}{cc} Drug & dissolution\;\left(\%\right)\\ & \;=\frac{Area\;(test)}{Area\;(standard)}\times \frac{Dissolution\;volume}{Label\;claim}\times Concentration\;standard\;\times \frac{Purity}{100}\\ & \;\times \frac{\left(100-water\;content\right)}{100}\times Dilution\;factor\times 100 \end{array}$$

#### 2.2.4. Preparation of Modified Solid Dispersions

Based on the evaluation results for the initial tablet formulations, modified solid dispersion (SD) batches (S17 to S32) were formulated to enhance their performance. The modification involved incorporating Polyplasdone^®^ XL10 as a disintegrant in varying concentrations in the polymeric solution with Kollidon^®^ VA64 [29]. The preparation steps followed the same co-precipitation process as for the main SDs, as mentioned in Section 2.2.1. The detailed compositions of these modified formulations (S17 to S32) are presented in Table 3.

#### 2.2.5. Preparation of Film-Coated Tablets Containing the Modified Solid Dispersions

Modified SDs (S17 to S32) were compressed into film-coated tablets (FCTs) (F17 to F32) and were prepared by the same process as for the main FCTs (F1 to F16).

Formulae F17 to F28 were compressed using a 11.0 mm round and plain punch, while formulae F29 to F32 were compressed on an 18.0 mm oblong, biconcave, plain punch.

The core tablets were coated using the same coating materials as mentioned before. The compositions of the new tablets are summarized in Table 4.

#### 2.2.6. Characterization of the Modified Film-Coated Tablets

The modified FCTs were evaluated for all pre-compression and post-compression parameters as mentioned for the main FCT evaluation in Section 2.2.2.

#### 2.2.7. Studying the Effect of Hardness on Dissolution Behavior

The F30 powder was compressed at a higher hardness limit, 12 KP, than the target limit (5–8 KP), and the new compressed formula was called F30*. The F30* formula was prepared via the same preparation method as F30 and compressed with the same punch but at a higher hardness value than F30.

#### 2.2.8. Criteria for Selection of the Optimized FCT Formula

##### Baseline Formula FCT Preparation for Comparative Evaluation

Both formulae F33 and F34 were prepared by the direct mixing method. The F33 formula was a physical mixture (PM) of the F30 formula that contained all materials of the F30 formula but the SD technique was not applied, and the F34 formula (control formula) contained all ingredients of the F30 formula except Kollidon^®^ VA64 [30], as shown in Table 5.

The F33 formula, BA, EZT, Kollidon^®^ VA64, Polyplasdone^®^ XL10, Kollidon^®^ CL, and Avicel^®^ PH 102 were sieved at mesh size 450 µm (#40 mesh). The blend was mixed manually for 10 min. Then, Aerosil^®^ 200 was also sieved at mesh size 250 µm (#60 mesh) and added to the previous blend. The blend was mixed manually for 5 min; finally, magnesium stearate was sifted at mesh size 250 µm (#60 mesh) and was added and mixed with the previous blend for 5 min [21]. The F34 formula was prepared via the same process as for the F33 formula but lacked the Kollidon^®^ VA64 polymer.

The final blend of both formulae was compressed into a tablet using the same compression machine as mentioned for the main FCTs using an 18.0 mm oblong, biconcave, plain punch.

Both formulae’s core tablets were coated using the same coating materials, the same ratio, and the same coating machine as mentioned for the main FCTs.

##### Baseline Formula FCT Characterization

Both formulae’s FCTs were evaluated for all pre-compression and post-compression parameters mentioned for the main FCT evaluation.

##### Solid-State Characterization

1. Differential Scanning Colorimetry (DSC)

DSC was conducted for BA, EZT, Kollidon^®^ VA64, Polyplasdone^®^ XL10, the physical mixture, and the SD30 part of the F30 formula to investigate the physical states of the drugs in the formulae. The DSC analysis was conducted on a Labsys Evo instrument using the gas option (TG-DSC 1200 °C) (SETARAM Instrumentation, Caluire-et-Cuire, France). Samples (13.87–21.63 mg) were placed into aluminum pans that were sealed with a lid. The thermograms were obtained at a heating rate of 10 °C/min from 0 °C to 450 °C [31].

2. X-Ray Powder Diffraction (XRPD)

XPRD was conducted to investigate the crystallinity of the drugs within the formulae. It was performed for BA, EZT, Kollidon^®^ VA64, Polyplasdone^®^ XL10, the physical mixture, and the SD30 part of the F30 formula. XPRD measurements were carried out using a X-ray diffractometer (Coupled Two Theta, Commander Sample ID system, Bruker AXS, Karlsruhe, Germany) set at 40 kV and 30 mA with CuKα radiation over a range of 2θ = 0–80° [32,33].

3. Fourier Transform Infrared Spectroscopy (FTIR)

The FTIR spectra of BA, EZT, Kollidon^®^ VA64, Polyplasdone^®^ XL10, the physical mixture, and the SD30 part of the F30 formula were investigated using a Bruker Alpha II ATR spectrometer (Bruker, Billerica, MA, USA) to study the interactions between the drugs and the carrier in solid dispersions through the KBr disc method. Samples were milled in an agate mortar, diluted with KBr powder, and compressed into transparent discs for FTIR examination. The spectra were collected over a range of 400 to 4000 cm^−1^ at a resolution of 2 cm^−1^ [34].

#### 2.2.9. Stability Study

F30 was blistered, packed in Alu/Alu packaging materials, and subjected to an accelerated stability study in a stability cabinet for up to six months according to the ICH guidelines [35,36] at 40 ± 2 °C with 75 ± 5% RH. Samples were withdrawn from the cabinet after six months of storage. They were physically inspected and analyzed for their drug content and in vitro dissolution profiles.

#### 2.2.10. In Vivo Evaluation of the Optimized Formulation

Twenty-five Sprague-Dawley rats weighing 150–200 g were obtained from the laboratory animal house of the Faculty of Veterinary Medicine, Zagazig University. Each rat was housed in a stainless-steel enclosure and was kept at 21–24 °C, offering a clean, pathogen-free environment. To achieve ideal living circumstances, the rats were exposed to a 12-h light–dark cycle and 60% relative humidity. All current experimental procedures were implemented following the ARRIVE guidelines for the use of laboratory animals and in vivo experiments and approved by the Institutional Animal Care and Use Committee (IACUC) of Zagazig University in Egypt (approval no.: ZU-IACUC/3/F/23/2023).

Rats were randomly distributed into five groups (*n* = 5); the first group served as a negative control (without induction of hyperlipidemia). The induction of hyperlipidemia in the other four rat groups was achieved via a Triton X-100 (Merck, Rahway, NJ, USA) with a single intraperitoneal injection of 100 mg/kg [37]. The five groups were classified as follows: Group I represented a normal group without the induction of hyperlipidemia; Group II represented hyperlipidemic rats who received no treatment (a positive control group); Group III represented hyperlipidemic rats who received an oral suspension of pure BA and EZT at a dose of 18 and 1 mg per kg of animal weight, respectively; Group IV received an oral suspension of Nexlizet^®^ in a dose equivalent to 18 and 1 mg/kg; Group V received an oral suspension of the developed formula (F30) in a dose equivalent to 18 and 1 mg/kg suspended in a phosphate buffer (pH 7.4). The dose of the combined BA and EZT was calculated by converting the human daily dose to the rat dose, AED (mg/kg) = human dose (mg/kg) × Km ratio [38]. All of the treated rat groups received the treatments for one week using oral gavage (Instech Laboratories Inc., Plymouth Meeting, PA, USA) [39].

Blood cholesterol levels, TC, TG, LDL, and HDL were measured using diagnostic kits (Spin React, Barcelona, Spain). Blood samples were collected from the rats’ lateral tail veins after their treatment according to the protocol mentioned before, where 1 mL of blood was collected from each rat by an investigator blinded to the treatment groups.

A one-way ANOVA followed by a Tukey post hoc test was used for multiple comparisons and applied to measure the statistical significance of the results using GraphPad Prism 8.0.1, San Diego, CA, USA. The level of significance was set as a *p*-value of <0.05.

## 3. Results and Discussion

### 3.1. Solid Dispersion Fabrication

The current study aimed at developing an optimized fixed-dose combination of BA/EZT as 180/10 mg film-coated tablets for the treatment of hypercholesterolemia without the incorporation of SLS (a surfactant-free formulation). Using the solid dispersion technique, different SD formulae were prepared using the Kollidon^®^ VA64 polymer in different ratios and utilizing different grades of disintegrant. First, our strategy depended on Kollidon^®^ CL as a disintegrant only; then, it was modified to utilize two different disintegrants (Kollidon^®^ CL/Polyplasdone^®^ XL10) to avoid the gelling effect problem associated with the solid dispersion technique, applying different ratios regarding the tablet weight to determine the disintegration time. This was considered the rate-limiting step in solid dispersion when formulated into tablet form due to the formation of a gel layer when the tablet comes into contact with water, which hinders water penetration into the tablet [25].

### 3.2. Film-Coated Tablet Characterization

#### 3.2.1. Preliminary Disintegration Time

Tablets were directly compressed to the target hardness limit of 5–8 KP (in-house specification) based on physical observations. As the D.T. is a crucial aspect in evaluating the performance of oral solid dosage forms, particularly those prepared by the solid dispersion technique, the test was initially performed as a preliminary evaluation to assess the basic performance of the different formulae before proceeding with the subsequent full characterization (pre- and post-compression evaluation). All prepared tablets were preliminary evaluated for the D.T., with a target limit of 300 s, to assess their performance and to guide the selection of the accepted formulae for further evaluation.

Regarding the main formulae (F1 to F16), they were designed with different ratios of polymer–disintegrant in order to evaluate their effects on disintegration behavior, with a target limit of 300 s, and the results are presented in Table 6. These formulae depended only on Kollidon^®^ CL as a disintegrant.

Formulae F1, F2, F3, and F4 demonstrated sufficient performance within the permissible hardness range (5–8 KP), complying with the D.T. standard. F1 was chosen for additional analysis because it demonstrated effective disintegration behavior (90 ± 10 s) with the lowest disintegrant concentration (5%) among them, suggesting that efficient wetting and matrix erosion facilitated rapid disintegration.

Among formulae F5, F6, F7, and F8, F5 did not meet the disintegration criteria, suggesting that either its formulation matrix hindered water penetration or there was insufficient porosity and the possibility of gel formation. However, F6, F7, and F8 were within the accepted limits, and F6 was selected due to its lower disintegrant ratio (10%), supporting the hypothesis that minimal yet effective disintegrant levels can achieve satisfactory performance if the formulation design supports rapid water uptake and matrix breakup [40].

From formulae F9 to F12, only F11 and F12 passed the D.T. test, with F11 chosen due to having the lowest disintegrant concentration among them (15%). The failure of F9 and F10 could be explained by the fact that the disintegrant ratio was not able to overcome the polymer’s gelling effect, leading to reduced water penetration and hindering matrix breakdown.

All formulations in the last group (F13 to F16) were out of specification, except for F16. The effective performance of F16 raises the possibility of a threshold beyond which disintegration may be negatively impacted by changing the excipient ratios or increasing the complexity of the formulation.

It was physically observed that all accepted formulations showed disintegration primarily through erosion rather than swelling or wicking mechanisms [41]. This indicates that the formulation design favored surface disintegration due to gradual erosion in the dissolution medium, rather than rapid water uptake and matrix rupture due to Kollidon^®^ VA64 forming a viscous surface layer. Given the previous results, it is important to balance the disintegrant-to-polymer ratio to maximize the performance of the tablet.

There was a contrasting influence of the two formulation variables (polymer and disintegrant ratios) on the tablet D.T. Increasing the amount of Kollidon^®^ VA64 resulted in an obvious increase in the mean D.T., which indicates a hindering effect on tablet disintegration due to the matrix integrity so that it decreases water penetration. In contrast, increasing the level of Kollidon^®^ CL led to a substantial reduction in D.T.; this confirms that it acts as a super-disintegrant, generating sufficient disruptive forces to accelerate tablet breakup. The steep negative slope observed in the main effects plot suggests that Kollidon^®^ CL is the main factor controlling the disintegration performance within the studied range.

Regarding the modified formulae (F17 to F32), shown in Table 6, they were formulated to study the impact of the addition of Polyplasdone^®^ XL10 as a disintegrant during the solid dispersion manufacturing process on the dissolution rate, as it has a large surface area and smaller particle size, at an average of 27 µm [42], differing from Kollidon^®^ CL, with an average of 110–130 µm [43]. The small particle size of Polyplasdone^®^ XL10 provides a larger surface area that can effectively cover and interact with the solid dispersion matrix, promoting rapid water uptake, improved disintegration, and enhanced dissolution.

The experimental design involved varying the disintegrant ratio of Polyplasdone^®^ XL10 to Kollidon^®^ CL, namely 25%/75%, 50%/50%, 75%/25%, and 100%/0%, for each selected formula from the main tablet. In each subgroup of formulae [(F17–F20), (F21–F24), (F25–F28), and (F29–F32)], the two formulae with the lowest Polyplasdone^®^ XL10 content (F17, F18; F21, F22; F25, F26; F29, F30, respectively) met the D.T. limit, while those with higher Polyplasdone^®^ XL10 content (F19, F20; F23, F24; F27, F28; F31, F32) failed to achieve the target limit. We selected formulae F17, F21, F25, and F29 from each group that passed with the lowest modified ratio for further characterization and dissolution profile analysis.

The results showed that Polyplasdone^®^ XL10’s physicochemical properties could improve the disintegration behavior; however, overuse may have a negative impact on the disintegration performance [44,45], as a cohesive matrix may form during SD formation, preventing water penetration and delaying disintegration. Polyplasdone^®^ XL10 disintegrates via a dual mechanism, namely initial swelling and water absorption, followed by erosion, as it is known to swell when in contact with water, generating internal pressure that facilitates matrix breakdown before the erosion phase takes over, as observed in the modified formulae (F17–F32). This was in contrast to the main formulae (F1–F16), which disintegrated primarily via the erosion mechanism, as they only contained Kollidon^®^ CL as a disintegrant.

Overall, the results highlight the importance of the disintegrant type, concentration, and formulation distribution. A balanced strategy regarding Polyplasdone^®^ XL10/Kollidon^®^ CL is crucial for maximizing disintegration. Our formulation succeeds in eliminating the gelling effect associated with tablet-based solid dispersion preparation.

#### 3.2.2. Pre-Compression Evaluation

##### Powder Characterization

Formulae F1, F6, F11, F16, F17, F21, F25, F29, F30, F33, and F34 were evaluated for their flowability properties based on their corresponding bulk density, tapped density, angle of repose, compressibility index, and Hausner ratio, and the corresponding results are presented in Table 7. The results showed that there was an obvious variation in the flow properties based on the differences in the composition.

For the chosen formulae among the main ones (F1, F6, F11, F16), F16 had the best results, with the highest bulk density (0.50 ± 0.01 g/mL) and the lowest compressibility index (14 ± 2.0%) and Hausner ratio (1.16 ± 0.03), showing excellent flowability. The other formulae showed similar excellent powder flowability but with a higher compressibility index and Hausner ratio, remaining within the acceptable limits.

Regarding the selected formulae among the modified formulae (F17, F21, F25, F29), F29 showed the most satisfactory results, with the highest bulk density (0.43 ± 0.02 g/mL) and the lowest compressibility index (12 ± 2.0%) and Hausner ratio (1.13 ± 0.02), indicating excellent flowability. On the other hand, both formulae F17 and F21 showed a false positive result for flowability, as the flowability was based on tapping and not powder-free flow through the funnel method.

F30 was developed as a confirmatory formulation for F29. It showed the best powder characteristics overall among all prepared formulae, with the lowest angle of repose (25.8 ± 0.2°), compressibility index (9 ± 1.0%), and Hausner ratio (1.10 ± 0.01). These values strongly support the importance of the formulation strategy.

On the other hand, the baseline formulae (F33 and F34) showed significantly poorer flow properties. F33 had a low bulk density (0.28 ± 0.02 g/mL) and a high angle of repose (38.5 ± 0.5°), but the angle of repose was obtained via tapping as the powder did not pass the funnel test, so the result is considered a false positive, confirming the poor powder flowability [46]. F34, with the lowest bulk density (0.26 ± 0.02 g/mL) and the highest compressibility index (29 ± 3.0%), exhibited the worst flow characteristics among all tested formulae, confirmed by its Hausner ratio of 1.41 ± 0.02, suggesting high interparticle cohesion and attraction forces between particles, being outside the acceptable range for direct compression [46]. These formulations can be considered unacceptable due to poor flow and compressibility, resulting from the highly hygroscopic APIs with poor powder flowability [9], which require further processing.

The improved bulk density, flowability, and compressibility of F30 are attributed to particle engineering effects associated with the solid dispersion system; the incorporation of the drug within the polymeric carrier likely promoted the formation of more uniform and aggregated particles with an improved packing ability [47].

Overall, these results highlight the critical role of pre-compression flow characterization in the selection of a suitable formula for tablet manufacturing. F30 was the formula selected as it demonstrated excellent powder properties, being suitable for the direct compression process.

#### 3.2.3. Post-Compression Evaluation

##### Physical Evaluation

The core tablets of the different formulae (F1, F6, F11, F16, F17, F21, F25, F29, F30, F30*, F33, and F34) were evaluated physically for their mean weight, mean diameter, mean thickness, hardness, friability, and D.T. (s), and the results are shown in Table 8.

The core tablet showed acceptable weight variation results within the range of the target weight ± 5%, which was reflected in both API assays. The difference in tablet diameter was due to the different punches used for compression and the increase in the tablet weight due to the change in polymer ratio [48]. The tablet weight is directly proportional to the tablet thickness, so the change in punch from 11.0 mm to 18.0 mm was important to accommodate the increased tablet weight in order to adjust the tablet thickness so that it is acceptable among patients during the swallowing process. The friability results were not higher than the target limit of 1.0%, which indicated that the tablets could withstand handling and further processing such as coating.

To confirm F29’s D.T. results, F30 was compressed at the same hardness as F29 (6.9 ± 0.4 KP) and demonstrated excellent performance, with a remarkable decrease in D.T. to 60 ± 30 s—the lowest D.T.—in comparison to F29, whose D.T. was 120 ± 30. This demonstrates that the distribution of the disintegrant is very important, and F30 is a promising formula.

To study the impact of increasing the hardness on the disintegration time, and hence the tablet’s dissolution profile, F30 was compressed at the target limit (6.9 ± 0.4 KP), and F30*, with the same composition as F30, was compressed with an elevated compression force (12 ± 1 KP) and showed a significantly slower D.T. of 120 ± 20 s, demonstrating the inverse correlation between the tablet hardness and disintegration efficiency. This finding aligns with the understanding that excessive compaction may reduce the porosity and hinder water penetration [49].

To evaluate F30 and judge the solid dispersion performance, the baseline formulae (F33 and F34) were prepared; the disintegration times were 10 ± 3 s and 5 ± 2 s, respectively, which are lower than that of F30. These are excellent D.T. results and are due to the more accessible distribution of the disintegrant in the physical mixture. In contrast, in F30, the partial incorporation of the Polyplasdone^®^ XL10 disintegrant within the solid dispersion matrix may reduce the efficiency of disintegrant action. Moreover, the hydrophilic polymer used in the SD may form a gel-like layer upon hydration, which can further hinder water penetration and delay tablet disintegration to some extent. However, both formulae were not accepted because they could not be compressed automatically as in the other formulae. They could only be compressed manually due to fair powder flowability and could not achieve the tablet weight automatically due to the decreased bulk density. Moreover, their low hardness (6.5 KP and 5.5 KP, respectively) and higher friability (0.45% and 0.5%, respectively) indicate reduced mechanical integrity, which may limit their robustness during handling and storage.

The F30 formula possesses sufficient mechanical strength at 7 KP (~7 MPa) to withstand downstream processing, including coating, packaging, handling, and transportation.

The coated tablets exhibited acceptable weight gain of 2–3% of the core tablet weight, indicating the consistent deposition of the coating material across the tablet, which was reflected in the observed uniformity of the color distribution.

##### Tablet Performance

1. Drug content

The drug content of the selected formulae (F1, F6, F11, F16, F17, F21, F25, F29, F30, F30*, F33, and F34) was analyzed using the HPLC method for both BA and EZT, and the results are presented in Table 9. They show that all formulae fell within the accepted range (90–110%) [50]. The results demonstrate acceptable content uniformity, indicating homogeneous drug distribution and suggesting good process consistency, which may support its potential scalability.

F25 showed a slight decrease in the EZT result (96.5 ± 3.9%), and F30 showed a slight increase in the BA result (105.5 ± 2.4%); the slight variation in content uniformity may be due to slight segregation during the mixing process with other excipients prior to compression. The F33 and F34 results were within the accepted limits, but F34 showed a slightly wider range of variability in both APIs, which may be attributed to batch scale-up effects or sampling variations.

Overall, the results confirm that the formulae provide satisfactory drug content uniformity but require further control (mixing time and mixing speed) during development and scale-up to maintain consistency during future production processes.

2. In vitro dissolution test

Nexlizet^®^ and the selected formulae were analyzed using the HPLC method for both APIs to determine their dissolution profiles. The similarity factors (F2) between Nexlizet^®^ and the other prepared formulae were compared using the optimal formula for further characterization. If the dissolution percentage is more than 85% in a 15-min period, the dissolution profile is considered to indicate very rapid behavior [51] and there is no need for F2 calculation.

BA was analyzed in accordance with the FDA-recommended dissolution medium (phosphate buffer, pH 6.6), which mimics the intestinal environment, where BA predominantly dissolves and is absorbed [52]. BA is considered pH-dependent, and its solubility and dissolution increased as the pH increased over the various time intervals studied. EZT was analyzed in accordance with the FDA dissolution medium (pH 4.5 with 0.45% SLS). As EZT does not dissolve well in water (BCS Class II) [53] or at any pH level [54], SLS (a surfactant) was added to the acidic medium (pH 4.5) to mimic its dissolution in the gut when bile salts and surfactants are present [55]. During the assay and dissolution analysis, there was no observed potential for cross-interference, as the co-formulated drug did not interfere with the quantification of either compound. Each drug was analyzed at its characteristic wavelength under its dissolution conditions, where no overlapping absorption was observed [27].

The comparison between the different formulae was conducted based on the drug content regardless of the tablet weight, even though the total weight of the tablets varied among formulations. We calculated and accurately compared the drug release profiles, which expressed the percentage of drug released over time. The drug content test was performed prior to the dissolution profile analysis to judge the dissolution results. Sink conditions were maintained for all comparisons by ensuring that the dissolution medium volume was at least ten times the drug saturation solubility. In our study, the dissolution medium was determined by the FDA regulations for each drug, which ensured that the dissolution was not dependent on the solubility limits and avoided drug saturation in the medium, enabling a meaningful comparison across various formulations [56,57].

**Figure 3:**
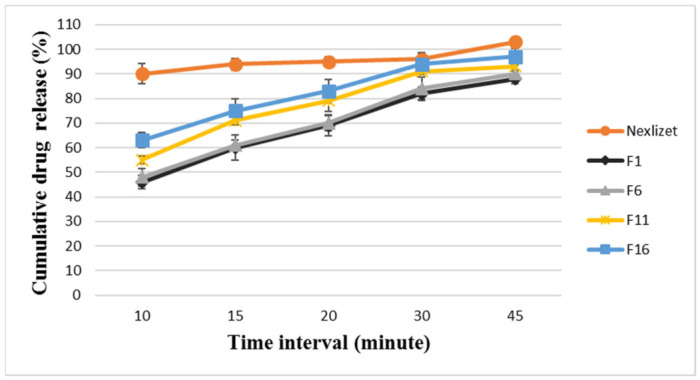
The dissolution profile results for BA in FDA-approved media at pH 6.6 for the formulae (Nexlizet 180/10 mg tablet lot number: 1750787; F1, F6, F11, and F16) at different time intervals (10, 15, 20, 30, and 45 min) ± SD (*n* = 6).

**Figure 4:**
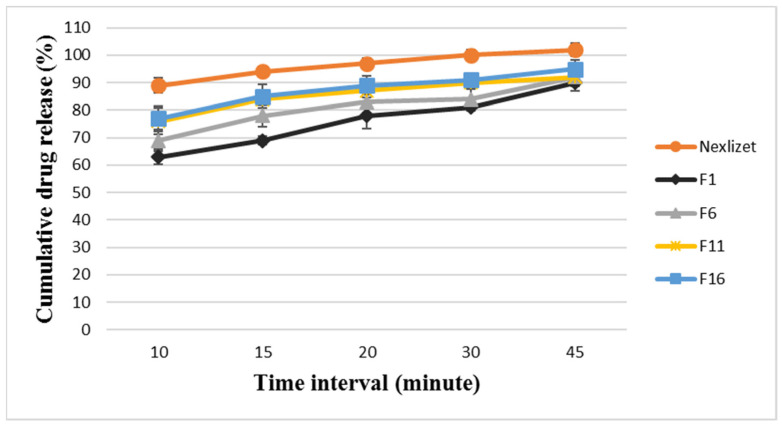
The dissolution profile results for EZT in FDA-approved media at pH 4.5 with sodium lauryl sulfate for the formulae (Nexlizet 180/10 mg tablet lot number: 1750787; F1, F6, F11, and F16) at different time intervals (10, 15, 20, 30, and 45 min) ± SD (*n* = 6).

Nexlizet^®^ and the selected main formulae (F1, F6, F11, and F16) were analyzed for their dissolution profiles for both APIs, and the results are summarized in Figure 3 and Figure 4. The results showed that Nexlizet^®^ displayed rapid release, with BA and EZT releasing around 94 ± 2.39% and 94 ± 2.04%, respectively, within 15 min and 103 ± 1.79% and 102 ± 2.39%, respectively, at 45 min; this indicates the immediate-release behavior of both APIs.

From the main formulae, F16 showed the best release profile, with BA and EZT releasing around 75 ± 4.9% and 85 ± 4.3%, respectively, at 15 min and 97 ± 3.49% and 95 ± 3.26% by 45 min. Although the delay in release was within 10 min, all formulae reached over 90% release by 45 min, complying with the general pharmacopeial requirements for dissolution but potentially falling short in terms of immediate-release performance or rapid therapeutic onset.

There is a proportional relation between the disintegrant concentration and dissolution profile, as the dissolution increased from F1 to F16, with F16 having the highest disintegrant concentration (20%).

Although the gelling effect of the SD technique was overcome by using Kollidon^®^ CL as a disintegrant at different ratios, the dissolution profile was not similar to that of Nexlizet^®^; this indicates that the formulation strategy using Kollidon^®^ CL alone was insufficient to enhance the dissolution profile in comparison with Nexlizet^®^. This was confirmed by the observations during the disintegration time test and the dissolution behavior in the different in vitro media, where the tablets disintegrated into large particles. Thus, modifications were applied to the formulation to decrease the disintegrating particles and to observe the effects on the dissolution profile.

The modification was achieved by the addition of Polyplasdone^®^ XL10 as a disintegrant through the solid dispersion phase preparation. This was smaller in particle size and was used in different ratios together with Kollidon^®^ CL as a disintegrant. The small particle size of Polyplasdone^®^ XL10 provides a larger surface area that can effectively cover and interact with the solid dispersion matrix, promoting rapid water uptake, improved disintegration, and enhanced dissolution.

**Figure 5:**
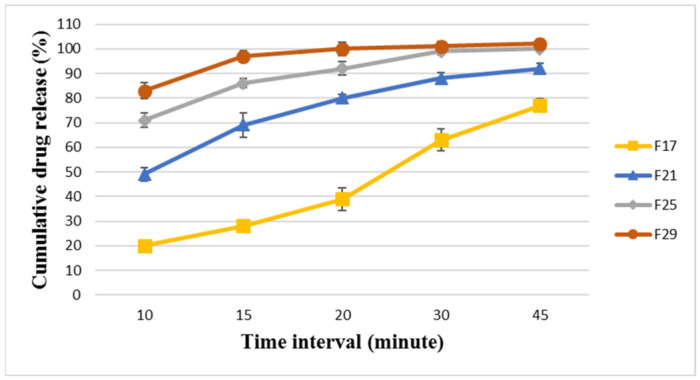
The dissolution profile results for BA in FDA-approved media at pH 6.6 for the formulae (F17, F21, F25, and F29) at different time intervals (10, 15, 20, 30, and 45 min) ± SD (*n* = 6).

**Figure 6:**
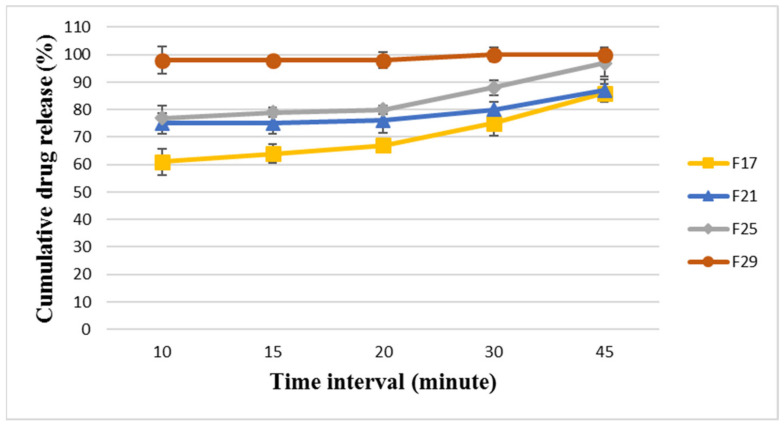
The dissolution profile results for EZT in FDA-approved media at pH 4.5 with sodium lauryl sulfate for the formulae (F17, F21, F25, and F29) at different time intervals (10, 15, 20, 30, and 45 min) ± SD (*n* = 6).

The selected modified formulae (F17, F21, F25, and F29) were analyzed for their dissolution profiles for both BA and EZT, and the results are presented in Figure 5 and Figure 6.

The results showed that F17 was the least releasing formula and showed delayed dissolution and release for BA and EZT, releasing around 28 ± 2.69% and 64 ± 3.57%, respectively, within 15 min and 77 ± 1.61% and 86 ± 3.29%, respectively, at 45 min. This indicates that the formula could not release its drug content even after 45 min on the dissolution apparatus; this is worse than the selected main formula that was released at around 90% after 45 min, and it failed to pass the pharmacopeial limit. The dissolution profile was enhanced from F17 to reach the optimum release for F29 with disintegrant type/ratio optimization.

F29 was the most promising formula, as it revealed the most rapid dissolution and release for BA and EZT, releasing around 97 ± 2.11% and 98 ± 1.65%, respectively, within 15 min and 102 ± 2.18% and 100 ± 2.79%, respectively, at 45 min. This means that it was the best optimized formulation among the modified formulae, providing rapid and complete dissolution for both APIs, which is essential in ensuring comparable in vitro dissolution and achieving bioequivalence with the reference product (Nexlizet^®^). Thus, the optimization of critical formulation parameters, such as the polymer ratio and disintegrant type and ratio, is essential.

**Figure 7:**
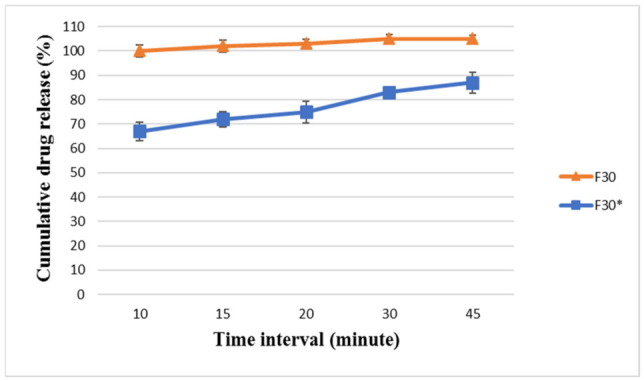
The dissolution profile results for BA in FDA-approved media at pH 6.6 for the formulae (F30 and F30*) at different time intervals (10, 15, 20, 30, and 45 min) ± SD (*n* = 6).

**Figure 8:**
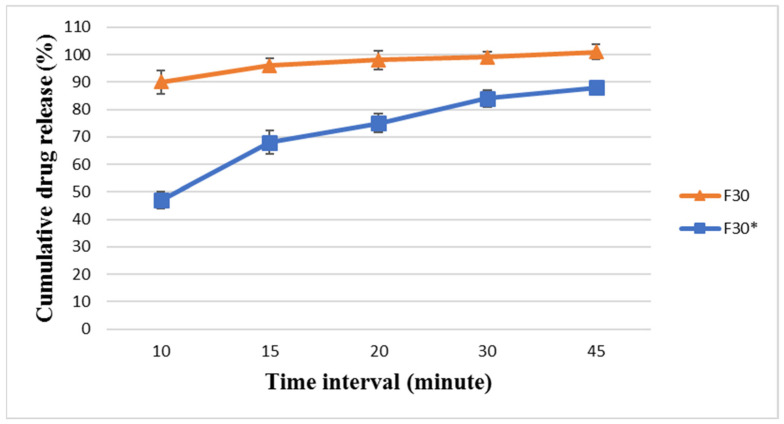
The dissolution profile results for EZT in FDA-approved media at pH 4.5 with sodium lauryl sulfate for the formulae (F30 and F30*) at different time intervals (10, 15, 20, 30, and 45 min) ± SD (*n* = 6).

F30 was developed as a confirmatory formulation for F29 to verify the dissolution profile trends and support the findings obtained with F29. F30 was analyzed, and the results are listed in Figure 7 and Figure 8. F30 showed very promising dissolution and release for BA and EZT, releasing around 102 ± 2.45% and 96 ± 2.51%, respectively, within 15 min and 105 ± 1.5% and 101 ± 2.7%, respectively, at 45 min. This is similar to Nexlizet^®^, as it released more than 85% of its drug content within 15 min. Although both F29 and F30 successfully passed the comparative dissolution analysis against Nexlizet^®^, F30 was selected as the optimized formulation for further studies due to its superior physicochemical properties, including improved flowability and compressibility. These results confirm that the formulation optimization successfully overcame the initial wetting limitations observed in earlier trials, leading to a rapid and complete release profile in the final formulation.

Although Nexlizet^®^ achieves rapid drug dissolution and release within 15 min, it is based on the presence of SLS to ensure adequate wetting and dissolution. In contrast, the F30 formula showed comparable dissolution behavior to Nexlizet without the use of SLS; this finding lies in the reduced formulation complexity and excipient burden. The F30 formula is considered SLS-free, which may potentially contribute to reduced BA variability. However, its clinical relevance, including any impact on patient tolerability, requires further investigation.

The F30 powder was also compressed to higher hardness (12 ± 1 KP) than the target limit (5–8 KP) to study the effect of hardness on tablet dissolution. The new formula with increased hardness, namely F30*, was analyzed. The results for F30* showed that the increase in hardness caused an obvious delay in the dissolution profile for both BA and EZT, releasing around 72 ± 3.2% and 68 ± 4.3%, respectively, within 15 min and 87 ± 4.2% and 88 ± 1.9%, respectively, at 45 min. The results are shown in Figure 7 and Figure 8. This indicates that hardness is a critical parameter influencing dissolution behavior, as it affects tablet porosity, disintegration, and the drug release rate. The hardness value is inversely proportional to dissolution.

**Figure 9:**
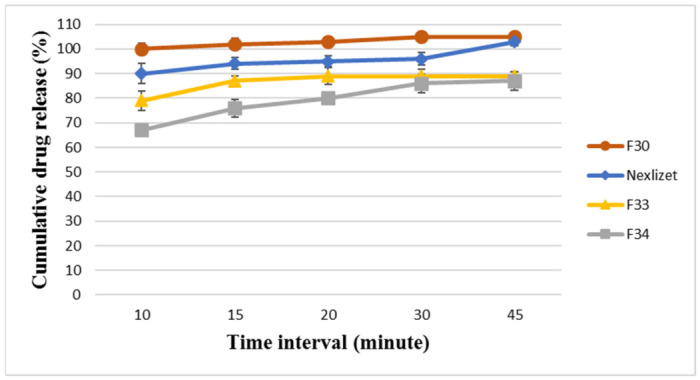
The dissolution profile results for BA in FDA-approved media at pH 6.6 for the formulae (F33, F34, F30, and Nexlizet 180/10 mg tablet lot number: 1750787) at different time intervals (10, 15, 20, 30, and 45 min) ± SD (*n* = 6).

**Figure 10:**
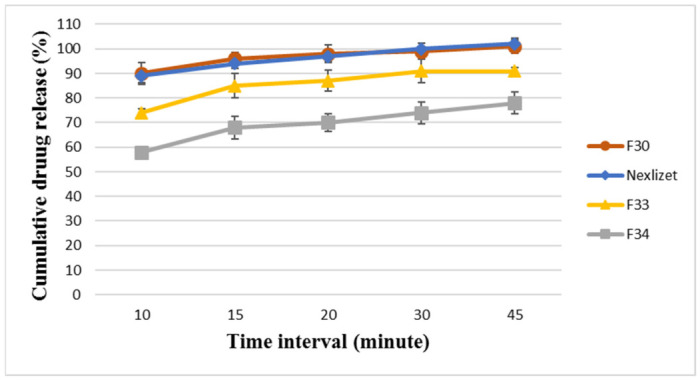
The dissolution profile results for EZT in FDA-approved media at pH 4.5 with sodium lauryl sulfate for the formulae (F33, F34, F30, and Nexlizet 180/10 mg tablet lot number: 1750787) at different time intervals (10, 15, 20, 30, and 45 min) ± SD (*n* = 6).

For the assurance of the solid dispersion performance in the F30 formula, the baseline formulae (F33 and F34) were analyzed, and the results are presented in Figure 9 and Figure 10. F33 showed a delay in dissolution and release compared to F30 for BA and EZT, releasing around 87 ± 2.04% and 85 ± 4.83%, respectively, within 15 min and 89 ± 89 ± 1.6% and 91 ± 1.34%, respectively, at 45 min. This means that the application of the solid dispersion technique plays a vital role in enhancing dissolution and release, as well as the physical parameters, such as powder flowability. F34 exhibited a greater delay in dissolution and release than F33 and F30 for BA and EZT, releasing around 76 ± 3.6% and 68 ± 4.64%, respectively, within 15 min and 87 ± 87 ± 3.82% and 78 ± 0.53%, respectively, at 45 min. This means that the incorporation of a hydrophilic polymer (Kollidon^®^ VA64) is very important for improving dissolution. Although the result for both APIs is about 100%, neither formula can release its content in 45 min, so the application of the solid dispersion technique (amorphization) is a promising strategy to enhance their water solubility.

#### 3.2.4. Solid Dispersion Characterization

To evaluate and confirm the performance of the optimized F30 formula and prove that the enhancement in the dissolution profiles of BA and EZT was due to the amorphous nature of the prepared SD, the solid-state (S30) part of the F30 formula was subjected to DSC, XPRD, and FTIR spectral studies to provide crucial information about the physical state, crystallinity, thermal behavior, and potential intermolecular interactions between the APIs and the carrier material.

##### Differential Scanning Colorimetry (DSC)

**Figure 11:**
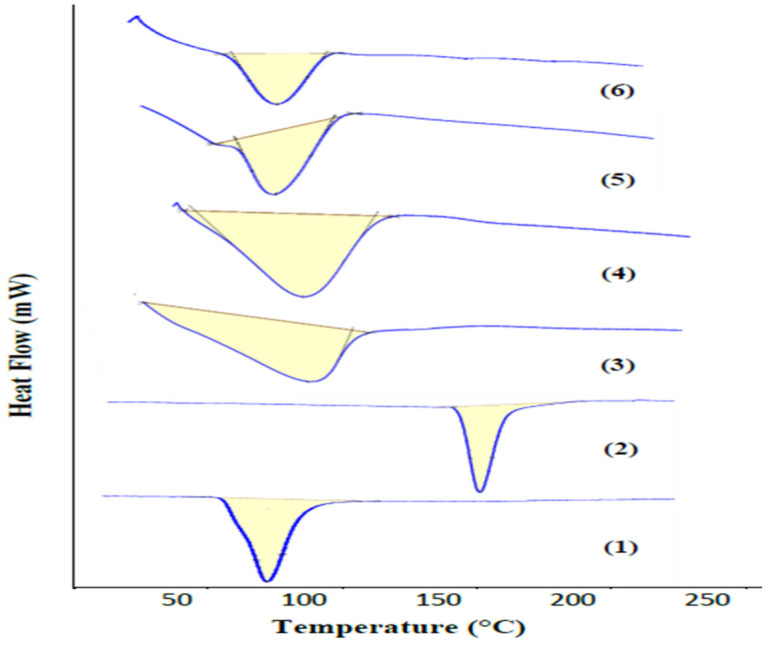
DSC curves of (1) bempedoic acid, (2) ezetimibe, (3) Kollidon^®^ VA64, (4) Polyplasdone^®^ XL10, (5) physical mixture (PM), and (6) S30 formula.

The thermo-analytical curves of BA, EZT, Kollidon^®^ VA64, Polyplasdone^®^ XL10, the physical mixture, and S30 are presented in Figure 11. The DSC thermogram of BA alone shows a sharp endothermic peak at 86–91 °C [58], related to its melting point. The DSC thermogram of EZT alone shows a sharp endothermic peak at 161–176 °C [59], related to its melting point. The DSC thermogram of Kollidon^®^ VA64 alone shows a broad endothermic peak with a maximum peak at 110 °C [59], related to the evaporation of the absorbed water by the polymer. The DSC thermogram of Polyplasdone^®^ XL10 alone shows a broad endothermic peak with a maximum peak at 115 °C, related to the evaporation of the water absorbed by the polymer. The peaks of both Kollidon^®^ VA64 and Polyplasdone^®^ XL10 are close each other due to their chemical relationship and are confirmed by the XRD pattern. The PM and S30 show endothermic peaks at 77–114 °C and 74–110 °C, respectively. The characteristic sharp peaks for BA and EZT are absent. The disappearance of the melting peak of BA may be due to overlapping polymer dehydration bands. In contrast, the disappearance of the EZT melting peak suggests possible dispersion efficiency in the hydrophilic polymer. The DSC thermograms of S30 and the PM were largely identical [60], and further FTIR and XRPD were required.

##### X-Ray Powder Diffraction (XRPD)

**Figure 12:**
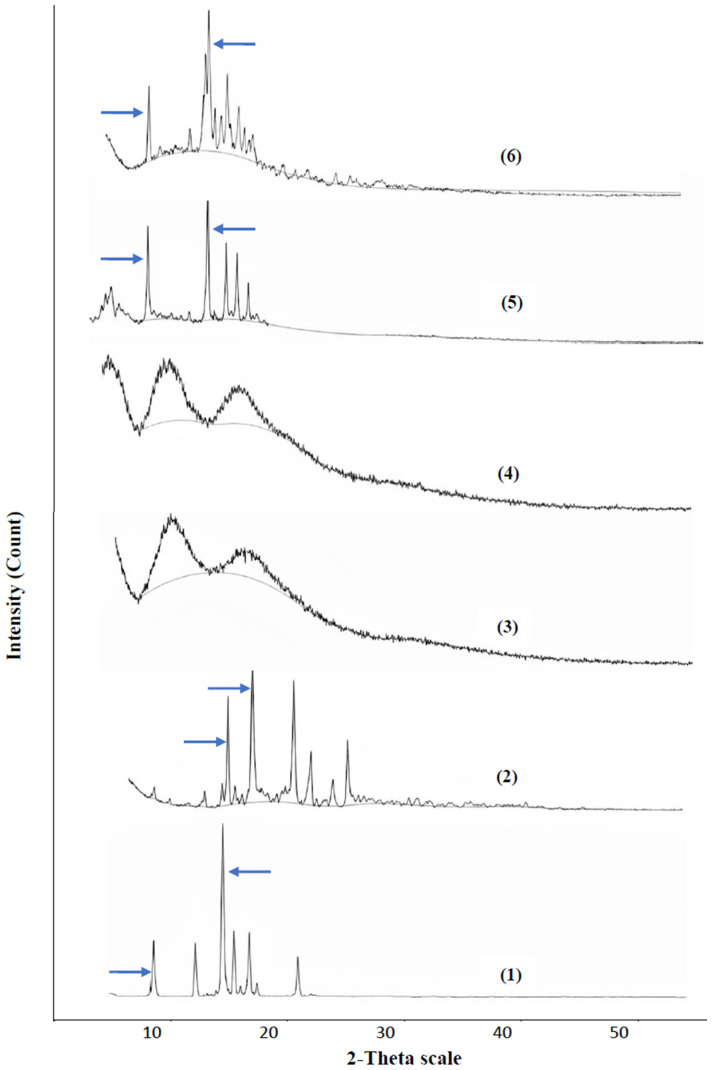
Powder X-ray diffraction patterns of (1) bempedoic acid, (2) ezetimibe, (3) Kollidon^®^ VA64, (4) Polyplasdone^®^ XL10, (5) physical mixture (PM), and (6) S30 formula.

The XRPD patterns of BA, EZT, Kollidon^®^ VA64, Polyplasdone^®^ XL10, the physical mixture, and S30 are presented in Figure 12. BA shows four distinctive, high diffraction peaks at 2 Ɵ values of 10.164° and 17.751° (±0.2 degree 2 theta) with intensities of 17,800 and 19,600, respectively, in addition to some other peaks with a lower intensity [58]. EZT shows a distinctive high diffraction peak pattern at 2 Ɵ values of 18.546° and 20.114° (±0.2 degree 2 theta) with intensities of 9300 and 10,400, respectively, in addition to some other peaks with a lower intensity [59]. Kollidon^®^ VA64 and Polyplasdone^®^ XL10 show only a few peaks with very weak intensities, indicating their amorphous nature [59].

The physical mixture revealed a sharp endothermic peak with a lower intensity and at 2 Ɵ values of 10.189° and 17.777° (±0.2-degree 2 theta) with intensities of 7800 and 8300, respectively, in addition to some other peaks with a lower intensity, and this suggests an amorphous state when mixed with polymers. Finally, the DSC of S30 showed a sharp endothermic peak with a lower intensity regarding the pure drugs at 2 Ɵ values of 10.379° and 17.991° (±0.2 degree 2 theta) with intensities of 3850 and 5900, respectively, due to the change in its crystalline nature to an amorphous state as a result of their incorporation into the S30 formula. The XRPD findings reveal the validity of solid dispersion as a technique to produce drugs in a reduced crystallinity state, and the observed improvement in dissolution is due to a number of factors, such as enhanced wettability and drug dispersion within the polymer matrix.

##### Fourier Transform Infrared Spectroscopy (FTIR)

**Figure 13:**
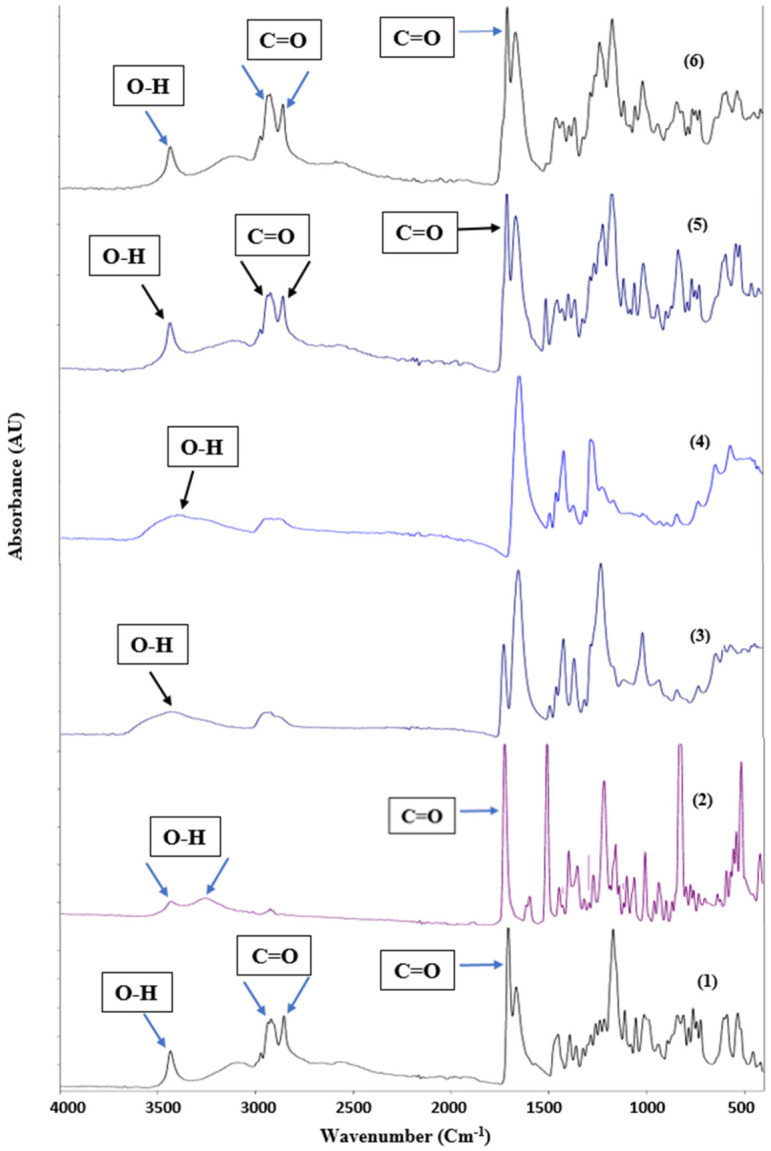
FTIR spectra of (1) bempedoic acid, (2) ezetimibe, (3) Kollidon^®^ VA64, (4) Polyplasdone^®^ XL10, (5) physical mixture (PM), and (6) S30 formula.

FTIR was performed to check the presence of chemical interactions between the APIs and the other excipients in the S30 formula. Figure 13 shows the FTIR spectra of pure BA, EZT, Kollidon^®^ VA64, Polyplasdone^®^ XL10, and S30.

The FTIR spectra of BA show characteristic peaks at 3443 (O-H), 2918, 2854, and 1710 (C=O) [61]. EZT’s FTIR spectra show strong absorption peaks from 3300 to 3400 cm^−1^ (intermolecular hydrogen-bonded O-H stretch), 1735 cm^−1^ (lactam ring C=O stretch), 1400 to 1600 cm^−1^ (aromatic C=C stretch), 1220 cm^−1^ (C-F stretch), 834 cm^−1^ (ring vibration due to para-substituted benzene), and 2917 cm^−1^ (sp^3^ C-H stretch) [62,63]. Kollidon^®^ VA 64 and Polyplasdone^®^ XL10 show broad peaks at 3443 cm^−1^ and 3393 cm^−1^, respectively [59]. The PM and S30 FTIR spectra show that the peaks of both drugs appear at the same position. This means that both APIs are dispersed into the polymer matrix and there is no major chemical interaction.

#### 3.2.5. Stability

Formula F30 was subjected to accelerated stability studies according to the ICH guidelines at 40 ± 2 °C/75 ± 5% RH for a period of 6 months. The formula was packed in Alu/Alu packaging material during the study. After 6 months, both APIs were evaluated for their drug content (assay) [accepted specification: not more than (NMT) 5% decrease from the starting drug content percentage] [64] and dissolution profiles [accepted specification: not less than (NLT) 80% drug release at a 30-min time interval] [9]. The results in terms of drug content are presented in Table 10, which indicates that the content of both APIs is within the accepted specification. The results regarding the dissolution profile were calculated as follows: for BA, it was 97 ± 2.91%, and, for EZT, it was 91 ± 0.88%. This indicates the release of both APIs within the accepted specification. Thus, F30 passed the stability test and is suitable for scaling up.

The dissolution profile of EZT was decreased to around 10%, which indicates that EZT gained some of its crystallinity due to the harsh stability conditions, but it is still within the accepted specification as mentioned for Nexlizet^®^ [9].

#### 3.2.6. In Vivo Antihyperlipidemic Evaluation of Optimized Formulation

**Figure 14:**
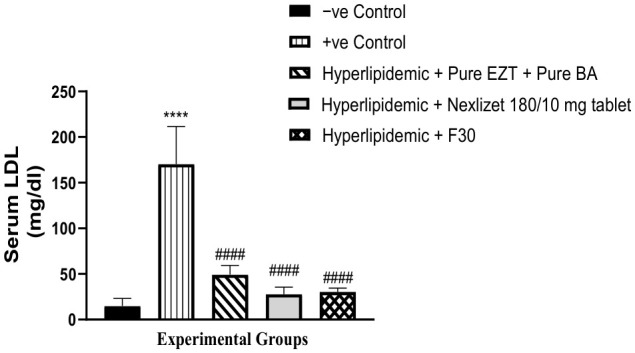
Serum LDL values are presented as mean (mg/dL) ± SEM, *n* = 5 per group. Data are presented with statistical significance indicated as **** (*p* < 0.0001; Group I vs. Group II), #### (*p* < 0.0001; Group II vs. Group III, Group IV and Group V).

As shown in Table 11 and Figure 14, we compared the ability of Group III (treated with pure BA/EZT), Group IV (treated with marketed tablets, Nexlizet^®^ 180/10 mg tablets), and Group V (treated with the selected formula, F30) to decrease LDL serum levels with that of the positive control Group II. A statistically significant decrease (*p* < 0.0001) was found for all treated groups. When comparing these groups (III, IV, and V) to the negative control group (I), the result was not statistically significant for any group, with *p*-values of 0.0791, 0.8330, and 0.7336, respectively, which indicated the ability of the groups to decrease LDL serum to the normal level. On comparing Group V with Group IV, the result was not statistically significant, with a *p*-value of 0.9847.

**Figure 15:**
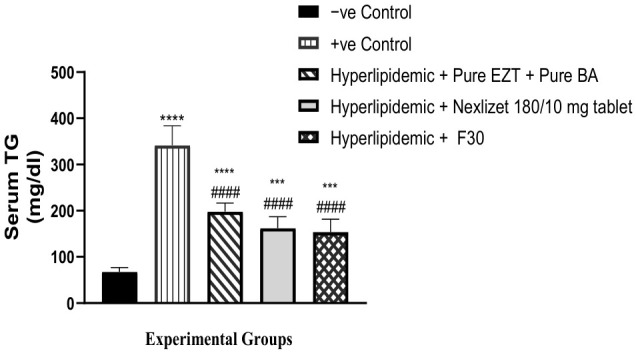
Serum TG values are presented as mean (mg/dL) ± SEM, *n* = 5 per group. Data are presented with statistical significance indicated as **** (*p* < 0.0001; Group I vs. Group II and Group III), *** (*p* < 0.001; Group I vs. Group IV and Group V), #### (*p* < 0.0001; Group II vs. Group III, Group IV and Group V).

As presented in Table 11 and Figure 15, we also compared the ability of Group III (treated with pure BA/EZT), Group IV (treated with marketed tablets, Nexlizet^®^ 180/10 mg tablets), and Group V (treated with the selected formula, F30) to decrease TG serum levels with that of the positive control Group II. A statistically significant decrease (*p* < 0.0001) was found for all treated groups. Comparing these groups (III, IV, and V) to the negative control group (I), the result was statistically significant for all groups, with *p*-values of >0.0001, 0.0002, and 0.0005, respectively, which indicated the inability of the groups to decrease TG serum to the normal level. When comparing Group V to Group IV, the result was not statistically significant, with a *p*-value of 0.9847.

**Figure 16:**
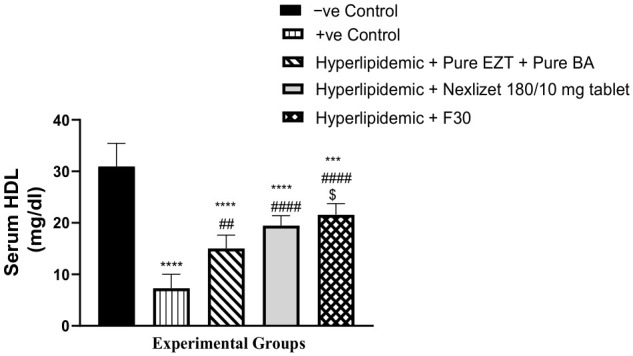
Serum HDL values are presented as mean (mg/dL) ± SEM, *n* = 5 per group. Data are presented with statistical significance indicated as **** (*p* < 0.0001; Group I vs. Group II, Group III and Group IV), *** (*p* < 0.001; Group I vs. Group V), ## (*p* < 0.01; Group II vs. Group III), #### (*p* < 0.0001; Group II vs. Group IV and V), and $ (*p* < 0.05; Group III vs. Group V).

As shown in Table 11 and Figure 16, we then compared the ability of Group III (treated with pure BA/EZT), Group IV (treated with marketed tablets, Nexlizet^®^ 180/10 mg tablets), and Group V (treated with the selected formula, F30) to decrease TC serum levels with that of the positive control Group II. A statistically significant decrease (*p* < 0.0001) was found for all treated groups. These results suggest that the formulations tested, particularly the selected formula F30, may offer effective alternatives for managing total cholesterol levels. Further studies are warranted to explore the long-term effects and potential mechanisms behind these outcomes. On comparing these groups (III, V, and IV) to the negative control group (I), the result was not statistically significant in Groups V and IV, with *p*-values of 0.4083 and 0.2619, respectively, but it was statistically significant in Group III, with a *p*-value of 0.0070. This indicates that Group III was unable to decrease TC serum to the normal level, but Groups V and IV did. On comparing Group V to Group IV, the result was not statistically significant, with a *p*-value of 0.9979.

**Figure 17:**
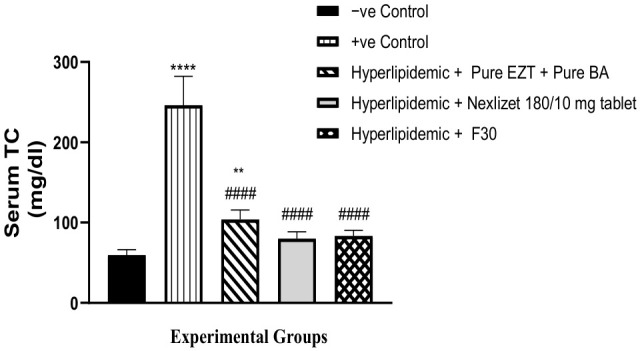
Serum TC values are presented as mean (mg/dL) ± SEM, *n* = 5 per group. Data are presented with statistical significance indicated as **** (*p* < 0.0001; Group I vs. Group II), ** (*p* < 0.01; Group I vs. Group III), #### (*p* < 0.0001; Group II vs. Group III, Group IV and Group V).

As presented in Table 11 and Figure 17, we next compared the ability of Group III (treated with pure BA/EZT), Group IV (treated with marketed tablets, Nexlizet^®^ 180/10 mg tablets), and Group V (treated with the selected formula, F30) to increase HDL serum levels with that of the positive control Group II. A statistically significant increase (*p* < 0.0001) was found for all treated groups. These findings suggest that both the selected formula and the marketed tablets are effective in enhancing HDL serum levels, indicating their potential utility in managing lipid profiles. Further research could explore the underlying mechanisms driving these increases and assess the long-term benefits of these treatments. On comparing these groups (III, IV, and V) to the negative control group (I), the result was statistically significant in all groups, with *p*-values of >0.0001, >0.0001, and 0.0004, respectively, which indicated the inability of all groups to decrease HDL serum to the normal level. On comparing the treated groups to each other, a statistically significant difference was found between Groups III and V, with a *p*-value of 0.0154, but no significant difference was observed, with a *p*-value of 0.7872, when Group V was compared to Group IV.

Finally, among all treated groups, Group III demonstrated the lowest treatment efficacy, which indicates that pure BA/EZT requires further modification to be used in the treatment of hyperlipidemia, regardless of wet granulation, as shown in Group IV, and the solid dispersion technique, as presented in Group V (F30). The results for the F30 formula were similar to those for the Nexlizet^®^ 180/10 mg tablet, which indicates that the newly developed formula (F30) is effective in the treatment of hyperlipidemia, as the results for F30 and Nexlizet^®^ were found to be not significant. These findings suggest that the new formulation could be a viable alternative to existing treatments, potentially offering similar efficacy (enhanced pharmacological performance) with different manufacturing advantages. Further pharmacokinetic studies may be necessary to explore the long-term effects and patient adherence to this treatment option.

## 4. Conclusions

The solid dispersion technique, specifically the co-precipitation method, is characterized by improved aqueous dissolution properties when formulating oral dosage forms intended for fixed-dose dyslipidemia treatment. The optimized polymer–disintegrant ratio showed a significant enhancement in drug dissolution in our study. A remarkable increase in the dissolution and release of both APIs was observed in the optimized F30 formula, prepared by solid dispersion, as compared with the baseline formulae (F33 and F34). The PXRD, DSC, and FTIR results confirmed that Kollidon^®^ VA64 inhibited drug crystallization, resulting in the drug’s amorphous state in the solid dispersion. The F30 formula demonstrated excellent physical properties and improved dissolution rates for both BA and EZT, successfully passed a comparative dissolution profile test against Nexlizet^®^, and completed an accelerated stability study lasting 6 months.

Our novel approach, F30, overcomes the disadvantages of the wet granulation technique with the absence of SLS (surfactant-free), mitigating the potential risk of BA variability during vivo administration. The F30 formula has the advantage of the preparation of the two APIs in one layer; this strategy allows the potential elimination of the weight variability between layers and potentially ensures better batch size control and improved content uniformity during manufacturing. The formula is lactose-free, in contrast to Nexlizet^®^, making it suitable for diabetic hyperlipidemic patients.

The in vivo study demonstrated a statistically significant decrease in the serum levels of TC, LDL, and TG in Group V, consisting of rats treated with the developed formula (F30), when compared with the positive control group (Group II), with a *p*-value of <0.0001, and a statistically significant increase (*p*-value of <0.0001) in the serum levels of HDL was recorded. All results regarding the lipid profile parameters for F30 and Nexlizet^®^ were found to be not significant, with a *p*-value of <0.0001. In conclusion, the F30 formula has a similar antihyperlipidemic effect to Nexlizet^®^.

## Figures and Tables

**Table 1:** Composition of the main SD part (S1 to S16).

Material		Function	Main Formula (mg)															
			S1	S2	S3	S4	S5	S6	S7	S8	S9	S10	S11	S12	S13	S14	S15	S16
Solid dispersion	Bempedoic acid	Active ingredient	180	180	180	180	180	180	180	180	180	180	180	180	180	180	180	180
	Ezetimibe	Active ingredient	10	10	10	10	10	10	10	10	10	10	10	10	10	10	10	10
	Kollidon^®^ VA64	Hydrophilic polymer	47.5	47.5	47.5	47.5	95	95	95	95	142.5	142.5	142.5	142.5	190	190	190	190

Each formula size was equivalent to the preparation of 100 tablets; the variation in SD weight was due to the difference in polymer ratio relative to the API ratio during formulation optimization.

**Table 2:** Compositions of the main formulae (F1 to F16) of film-coated tablets.

Preparation Phase	Material	Function	Main Formula (mg)															
			F1	F2	F3	F4	F5	F6	F7	F8	F9	F10	F11	F12	F13	F14	F15	F16
SD part (S1 to S16)			237.5	237.5	237.5	237.5	285	285	285	285	332.5	332.5	332.5	332.5	380	380	380	380
Excipient	Kollidon^®^ CL	Disintegrant	15.23	32.55	52.4	75.4	18.27	39.04	62.87	90.5	21.3	45.55	73.36	105.55	24.35	52.05	83.79	120.65
	Avicel^®^ PH 102	Filler	45.69	48.8	52.4	56.56	54.81	58.56	62.87	67.86	63.95	68.3	73.36	79.15	73.1	78.1	83.79	90.5
	Aerosil^®^ 200	Glidant	3.04	3.25	3.49	3.77	3.65	3.9	4.19	4.52	4.26	4.55	4.89	5.27	4.87	5.2	5.58	6.03
	Magnesium stearate	Lubricant	3.04	3.25	3.49	3.77	3.65	3.9	4.19	4.52	4.26	4.55	4.89	5.27	4.87	5.2	5.58	6.03
Total core tablet weight			304.5	325.35	349.28	377	365.38	390.4	419.12	452.4	426.27	455.45	489	527.74	487.19	520.55	558.74	603.21
Total coated tablet weight			310.6	331.86	356.27	384.5	372.69	398.2	427.50	461.4	434.79	464.56	498.7	538.29	496.93	530.96	569.91	615.27

Each formula size was equivalent to preparing 100 tablets; the variation in tablet weight among formulations was due to intentional changes in polymer and disintegrant ratios during formulation optimization. The relative proportions (ratios) of the remaining excipients were kept constant, although their absolute weight varied accordingly.

**Table 3:** Composition of the modified SD part (S17 to S32).

Material		Function	Modified Formula (mg)															
			S17	S18	S19	S20	S21	S22	S23	S24	S25	S26	S27	S28	S29	S30	S31	S32
Solid dispersion	Bempedoic acid	Active ingredient	180	180	180	180	180	180	180	180	180	180	180	180	180	180	180	180
	Ezetimibe	Active ingredient	10	10	10	10	10	10	10	10	10	10	10	10	10	10	10	10
	Kollidon^®^ VA64	Hydrophilic polymer	47.5	47.5	47.5	47.5	95	95	95	95	142.5	142.5	142.5	142.5	190	190	190	190
	Polyplasdone^®^ XL10	Disintegrant	3.81	7.62	11.42	15.23	9.76	19.52	29.28	39.04	18.34	36.68	55.02	73.36	30.16	60.33	90.49	120.65

Each formula size was equivalent to preparing 100 tablets; the variation in SD weight was due to the differences in polymer and disintegrant ratios during formulation optimization.

**Table 4:** Compositions of the modified formulae (F17 to F32) of film-coated tablets.

Preparation Phase	Material	Function	Modified Formula (mg)															
			F17	F18	F19	F20	F21	F22	F23	F24	F25	F26	F27	F28	F29	F30	F31	F32
SD part (S17 to S32)			241.31	245.12	248.92	252.73	294.76	304.52	314.28	324.04	350.84	369.18	387.52	405.86	410.16	440.33	470.49	500.65
Excipient	Kollidon^®^ CL	Disintegrant	11.42	7.615	3.81	-	29.28	19.52	9.76	-	55.02	36.68	18.34	-	90.49	60.32	30.16	-
	Avicel^®^ PH 102	Filler	45.69	45.69	45.69	45.69	58.56	58.56	58.56	58.56	73.36	73.36	73.36	73.36	90.5	90.5	90.5	90.5
	Aerosil^®^ 200	Glidant	3.04	3.04	3.04	3.04	3.9	3.9	3.9	3.9	4.89	4.89	4.89	4.89	6.03	6.03	6.03	6.03
	Magnesium stearate	Lubricant	3.04	3.04	3.04	3.04	3.9	3.9	3.9	3.9	4.89	4.89	4.89	4.89	6.03	6.03	6.03	6.03
Total core tablet weight			304.5	304.5	304.5	304.5	390.4	390.4	390.4	390.4	489	489	489	489	603.21	603.21	603.21	603.21
Total coated tablet weight			310.5	310.59	310.59	310.59	398.21	398.21	398.21	398.21	434.79	434.79	434.79	434.79	615.27	615.27	615.27	615.27

Each formula size was equivalent to preparing 100 tablets; the variation in tablet weight among formulations was due to intentional changes in polymer and disintegrant ratios during formulation optimization. The relative proportions (ratios) of the remaining excipients were kept constant, although their absolute weight varied accordingly.

**Table 5:** Compositions of the baseline formulae F33 and F34 as film-coated tablets.

Formula	Component (in mg)/Function								Total Core Tablet Weight	Total Coated Tablet Weight
	Bempedoic Acid	Ezetimibe	Kollidon^®^ VA64	Polyplasdone^®^ XL10	Kollidon^®^ CL	Avicel^®^ PH 102	Aerosil^®^ 200	Magnesium Stearate		
	Active Ingredient	Active Ingredient	Hydrophilic Polymer	Disintegrant	Disintegrant	Filler	Glidant	Lubricant		
F33	180	10	190	60.325	60.325	90.5	6.03	6.03	603.21	615.27
F34	180	10	-	60.325	60.325	280.5	6.03	6.03	603.21	615.27

Each formula size was equivalent to preparing 100 tablets; these formulae are equivalent to the F30 formula.

**Table 6:** The results regarding the D.T. (s) for formulae F1 to F32.

Category	Formula Code	Disintegration Time D.T. (s) ± SD
Main Formulae	F1	90 ± 10
	F2	20 ± 5
	F3	15 ± 5
	F4	10 ± 5
	F5	540 ± 30
	F6	140 ± 20
	F7	20 ± 5
	F8	15 ± 5
	F9	More than 900
	F10	840 ± 30
	F11	50 ± 10
	F12	40 ± 5
	F13	More than 900
	F14	600 ± 45
	F15	360 ± 30
	F16	100 ± 30
Modified Formulae	F17	100 ± 30
	F18	80 ± 30
	F19	540 ± 120
	F20	>900
	F21	110 ± 20
	F22	60 ± 30
	F23	540 ± 120
	F24	>900
	F25	110 ± 30
	F26	50 ± 30
	F27	540 ± 120
	F28	>900
	F29	120 ± 30
	F30	60 ± 30
	F31	540 ± 120
	F32	>900

Data are presented as mean ± SD (*n* = 6).

**Table 7:** Pre-compression evaluation of F1, F6, F11, F16, F17, F21, F25, F29, F30, F33, and F34 tablets.

		Parameter					
Category	Formula Code	Bulk Density (g/mL) ± SD	Tapped Density (g/mL) ± SD	Angle of Repose (°) ± SD	Compressibility Index (%) ± SD	Hausner Ratio (%) ± SD	Flow
Selected from Main Formulae	F1	0.36 ± 0.02	0.44 ± 0.01	29.1 ± 0.4	19 ± 1.0	1.24 ± 0.02	Excellent
	F6	0.43 ± 0.01	0.51 ± 0.01	28.5 ± 0.5	17 ± 2.0	1.20 ± 0.03	Excellent
	F11	0.44 ± 0.02	0.52 ± 0.03	28.3 ± 0.6	15 ± 1.0	1.18 ± 0.01	Excellent
	F16	0.5 ± 0.01	0.58 ± 0.03	27.7 ± 0.5	14 ± 2.0	1.16 ± 0.03	Excellent
Selected from Modified Formulae	F17	0.38 ± 0.01	0.45 ± 0.02	29.3 ± 0.5 Tapping	21 ± 1.0	1.27 ± 0.08	Excellent
	F21	0.39 ± 0.01	0.48 ± 0.01	28.1 ± 0.5 Tapping	19 ± 3.0	1.23 ± 0.06	Excellent
	F25	0.39 ± 0.02	0.47 ± 0.02	26.4 ± 0.4	16 ± 1.0	1.19 ± 0.01	Excellent
	F29	0.43 ± 0.02	0.48 ± 0.02	27.2 ± 1.1	12 ± 2.0	1.13 ± 0.02	Excellent
Confirmatory Formula	F30	0.43 ± 0.02	0.47 ± 0.03	25.8 ± 0.2	9 ± 1.0	1.10 ± 0.01	Excellent
Baseline Formulae	F33	0.28 ± 0.02	0.47 ± 0.03	38.5 ± 0.5 Tapping	27 ± 1.0	1.10 ± 0.01	Poor
	F34	0.26 ± 0.02	0.37 ± 0.01	35 ± 0.4 Tapping	29 ± 3.0	1.41 ± 0.02	Poor

Sample size (20 g of the prepared powder); data are presented as mean ± SD (*n* = 3).

**Table 8:** Post-compression evaluation of F1, F6, F11, F16, F17, F21, F25, F29, F30, F30*, F33, and F34 tablets.

Category	Formula Code	Parameter					
		Mean Weight (mg) ± SD	Mean Diameter (mm) ± SD	Mean Thickness (mm) ± SD	Hardness (KP) ± SD	Friability (%) ± SD	D.T. (s) ± SD
Selected from Main Formulae	F1	307.45 ± 7.0	10.96 ± 0.03	4.12 ± 0.03	6.5 ± 0.5	0.41 ± 0.07	90 ± 10
	F6	395.73 ± 10.0	11.1 ± 0.05	5.11 ± 0.05	7.6 ± 0.4	0.39 ± 0.09	140 ± 20
	F11	495.35 ± 11.0	10.89 ± 0.02	5.78 ± 0.04	6.6 ± 0.2	0.36 ± 0.04	50 ± 10
	F16	604.98 ± 15.0	18.31 ± 0.01	5.79 ± 0.03	6.3 ± 1.0	0.30 ± 0.02	100 ± 30
Selected from Modified Formulae	F17	309.25 ± 9.0	10.98 ± 0.05	4.15 ± 0.02	6.5 ± 0.9	0.45 ± 0.05	100 ± 30
	F21	396.47 ± 12.0	11.08 ± 0.03	5.14 ± 0.03	6.9 ± 0.5	0.41 ± 0.09	110 ± 20
	F25	492.76 ± 10.0	10.94 ± 0.06	5.83 ± 0.02	7.1 ± 0.7	0.32 ± 0.02	110 ± 30
	F29	600.91 ± 12.0	18.28 ± 0.01	5.89 ± 0.01	6.4 ± 0.9	0.2 ± 0.05	120 ± 30
Confirmatory Formulae	F30	605.27 ± 9.5	18.1 ± 0.02	5.54 ± 0.04	6.9 ± 0.4	0.26 ± 0.07	60 ± 30
	F30*	604.41 ± 7.5	17.88 ± 0.12	5.16 ± 0.02	12 ± 1.0	0.12 ± 0.05	120 ± 20
Baseline Formulae	F33	607.11 ± 8	18.28 ± 0.04	6.1 ± 0.02	6.5 ± 1	0.45 ± 0.05	10 ± 3
	F34	595.14 ± 12	18.14 ± 0.04	5.34 ± 0.03	5.5 ± 2	0.5 ± 0.02	5 ± 2

For mean weight, mean diameter, mean thickness, and hardness (1 KP~MPa), data are presented as mean ± SD (*n* = 10). For D.T., data are presented as mean ± SD (*n* = 6). For friability, sample size was 6.5 g of tablet and data are presented as mean ± SD (*n* = 3).

**Table 9:** Drug content results for F1, F6, F11, F16, F17, F21, F25, F29, F30, F30*, F33, and F34 tablets.

Category	Formula Code	Drug Content %	
		Bempedoic Acid	Ezetimibe
Selected from Main Formulae	F1	99.3 ± 2.4	100.6 ± 1.9
	F6	103.42 ± 1.4	98.2 ± 3.5
	F11	98.4 ± 3.5	102.4 ± 1.2
	F16	99.7 ± 3.1	97.5 ± 2.8
Selected from Modified Formulae	F17	97.11 ± 2.2	98.7 ± 3.3
	F21	101.4 ± 1.8	99.4 ± 1.5
	F25	98.7 ± 1.2	96.5 ± 3.9
	F29	100.1 ± 1.9	99.7 ± 1.8
Confirmatory Formulae	F30	105.5 ± 2.4	101.5 ± 2.1
	F30*	99.4 ± 1.5	99.8 ± 1.7
Baseline Formulae	F33	98.3 ± 2.7	100.9 ± 2.9
	F34	97.1 ± 3.4	97.9 ± 3.7

Data are presented as mean ± SD (*n* = 6).

**Table 10:** Drug content % after accelerated stability study.

Time of Analysis (Months)	Drug Content % ± SD	
	Bempedoic Acid	Ezetimibe
0	107.58 ± 0.51	101.74 ± 1.6
6	105.79 ± 1.46	98.62 ± 1.12

Data are presented as mean ± SD (*n* = 6).

**Table 11:** Serum blood levels of TC, TG, HDL, and LDL in different rat groups.

Group	TC (mg/dL)	TG (mg/dL)	LDL (mg/dL)	HDL (mg/dL)
Group I	59.49 ± 3.00	67.91 ± 3.99	14.92 ± 3.68	30.99 ± 1.99
Group II	245.97 ± 16.22	341.31 ± 19.12	170.39 ± 18.40	7.31 ± 1.21
Group III	104.01 ± 5.27	198.09 ± 8.24	49.32 ± 4.46	15.06 ± 1.14
Group IV	79.86 ± 3.88	162.42 ± 10.94	27.89 ± 3.41	19.49 ± 0.85
Group V	83.35 ± 3.21	153.63 ± 12.43	30.25 ± 1.93	21.57 ± 0.98

Group I is a normal group (negative control), Group II represents hyperlipidemic non-treated rats (positive control), Group III represents rats treated with pure EZT and BA, Group IV represents rats treated with marketed tablets (Nexlizet 180/10 mg), and Group V represents rats treated with the selected formula (F30). Data are presented as (mg/dL ± standard error of mean, “SEM”) (*n* = 5).

## Data Availability

The original contributions presented in this study are included in the article. Further inquiries can be directed to the corresponding authors.
